# Cardiopulmonary exercise testing as a prognosis‐assessing tool in heart failure with preserved ejection fraction

**DOI:** 10.1002/ehf2.15219

**Published:** 2025-01-16

**Authors:** C. Rozados da Conceicao, A. Krannich, V. Zach, R. Pinto, A. Deichl, A. Feuerstein, L. Schleussner, F. Edelmann

**Affiliations:** ^1^ Department of Cardiology, Angiology and Intensive Care Medicine Deutsches Herzzentrum der Charité Berlin Germany; ^2^ Charité – Universitätsmedizin Berlin Berlin Germany; ^3^ DZHK (German Center for Cardiovascular Research) partner site Berlin Berlin Germany; ^4^ BioStats Berlin Germany; ^5^ Berlin Institute of Health Charité – Universitätsmedizin Berlin Berlin Germany; ^6^ Department for Internal Medicine and Cardiology, Heart Centre Dresden, Faculty of Medicine and University Hospital Carl Gustav Carus TUD Dresden University of Technology Dresden Germany

**Keywords:** CPET, HFpEF, Prognosis, Ventilatory inefficiency

## Abstract

**Aims:**

Patients with heart failure with preserved ejection fraction represent half of the heart failure patients nowadays, an at least steady trend due to the aging of the population. We investigated whether the parameters obtained from cardiopulmonary exercise testing (CPET) correlated with the prognosis of these patients. This prospective observational cohort study assesses the relationship between the CPET parameters peakVO_2_ and VE/VCO_2_ slope and the number of heart failure hospitalizations or cardiovascular death of these patients.

**Methods and results:**

From August 2016 until May 2019, 99 patients from our outpatient unit with newly diagnosed heart failure with preserved ejection fraction underwent CPET. Median follow‐up was 30 months [interquartile range, 24–38.5]. We selected peakVO_2_ < 14 mL/min/kg and a VE/VCO_2_ slope > 34 as threshold values for our primary clinically relevant endpoint, a composite of hospitalization for heart failure or cardiovascular death. Mean age was 75.07 ± 7.31 years, 49% were women, 75% were at NYHA class II and median NTproBNP was 511 pg/mL. Mean peakVO_2_ was 15.09 ± 4.75, and mean VE/VCO_2_ was 36.05 ± 6.60. During follow‐up, there were 207 all‐cause hospitalizations, 126 cardiovascular hospitalizations, 58 heart failure hospitalizations and 4 deaths. Over a median follow‐up of 30 months, the primary clinically relevant endpoint occurred in 5 of 40 patients (12.5%) with a VE/VCO_2_ slope ≤ 34 and in 19 of 59 patients (32.2%) with a VE/VCO_2_ slope > 34 [hazard ratio, 2.69; 95% confidence interval (CI), 1.00–7.21; *P* = 0.04]. On multivariate analysis, VE/VCO_2_ slope was independently associated with heart failure hospitalization or cardiovascular death as a terminal event.

**Conclusions:**

In patients with heart failure with preserved ejection fraction, a VE/VCO_2_ slope > 34 predicts heart failure hospitalizations and cardiovascular death.

## Introduction

Heart failure with preserved ejection fraction (HFpEF) accounts for half of the patients with heart failure and its prevalence increases with an aging population whereas its mortality remains unchanged, representing a growing public health problem.^1^ In comparison with HFrEF patients, patients with HFpEF are older and more often female. Also, comorbidities are more commonly found in patients with HFpEF than in patients with HFrEF.^2^ Population‐based data from hospitalized patients have shown similar prognostic outcomes in patients with HFpEF and HFrEF.^1^, ^3^

Exercise testing as a prognosis assessment tool in these patients is a promising strategy since exercise intolerance is the leading manifestation of heart failure. In heart failure with reduced ejection fraction (HFrEF), exercise testing has proven to identify patients for high risk of early death and therefore to deliver reliable results when prioritizing patients for heart transplantation.^4^ Besides peakVO_2_ and VE/VCO_2_ slope, tried and tested parameters in HFrEF, the existing literature proposed new parameters such as percentage of predicted peakVO_2_ or percent of predicted maximum oxygen uptake or a flattening oxygen consumption trajectory during CPET as indicators of a poorer prognosis.^5^, ^6^, ^7^

In contrast, to date, studies in HFpEF patients delivered diverging conclusions regarding the prognostic value of peakVO_2_ and VE/VCO_2_ slope, partly due to the recent definition of HFpEF, partly due to the retrospective character of these studies.^5^, ^8^, ^9^ The prognostic potential of cardiopulmonary exercise testing when combined with stress echocardiography (cardiopulmonary exercise testing‐exercise stress echocardiography, CPET‐ESE) has been recently proven in both HFpEF patients and subjects at risk of developing HF.^10^, ^11^

With the rise of the HFpEF^1^ as a drive, the harmonization of its diagnosis in the current guidelines^12^ as a precedent and the development of diagnostic algorithms as a role model,^13^, ^14^ the increasing interest in better prognostic tools for risk stratification in this population seemed only natural.

In this study, we prospectively analysed a cohort of patients with HFpEF that underwent CPET with regard to their risk of cardiovascular and heart failure hospitalization and death.

## Methods

### Study population

Between August 2016 and May 2019, consecutive patients with a diagnosis of HFpEF evaluated in an outpatient setting at the Charité University Hospital were prospectively enrolled in the German HFpEF Registry. Inclusion criteria were (1) New York Heart Failure (NYHA) functional class ≥ II, (2) LVEF ≥ 50%, (3) elevated levels of natriuretic peptides [NTproBNP > 125 pg/mL (for sinus rhythm) or >365 pg/mL (for atrial fibrillation)] and (4) at least one additional criteria for structural heart disease or diastolic dysfunction (LV mass index ≥ 115 g/m^2^ for males and ≥95 g/m^2^ for females; LA volume index >34 mL/m^2^; mean E/e′ ≥ 13 and mean e′ < 9 cm/s).^12^ Patients were excluded if they had more than moderate valve disease, significant mitral annular calcification, congenital heart disease, previous cardiac transplantation, acute coronary syndrome or cardiac surgery/percutaneous intervention during the past 3 months, restrictive cardiomyopathy, chronic obstructive pulmonary disease, severe kidney disease (eGFR_MDRD_ ≤ 30 mL/min/1.73m^2^ or requiring dialysis) and severe liver disease (Child–Pugh Class B and C or with indication for liver transplantation). The ethics committee of Charité University Hospital approved the research project, and written informed consent was obtained from all subjects.

### Clinical characteristics

The following data were collected in all study participants: demographics, body mass index, blood pressure, cardiovascular risk factors, comorbidities, clinical history, NYHA functional class, medications. An electrocardiogram and an echocardiography were performed at the time of the first clinical evaluation after enrolment. Blood samples were collected for laboratory testing, including haemoglobin, creatinine, HbA1c and NTproBNP. Study data were collected and managed using REDCap electronic data capture tools hosted at Charité University Hospital.^15^, ^16^

### Procedures

#### Cardiopulmonary exercise testing (CPET)

All patients underwent a symptom‐limited CPET using a cycle ergometer with minute‐by‐minute increments, starting at a workload of 20 W, followed by a 10 W increment every minute. Cycle ergometry was chosen and the exercise test protocol was tailored for our elderly cohort as per current recommendations.^17^ Heart rate was continuously monitored by electrocardiography at rest and during exercise; blood pressure was measured at rest and every 2 min. Breath‐by‐breath oxygen consumption (VO_2_), carbon dioxide production (VCO_2_) and minute ventilation (VE) were acquired and averaged over 10 s intervals using a ventilator expired gas analysis system. Analyses were performed using MetaSoft Studio version 5.12.2.0 (CORTEX Biophysik GmbH, Leipzig/Germany). Test termination occurred upon patients request due to symptoms or fulfilling any of the following criteria at any point of the test: evidence of ventricular arrhythmia, ST segment depression ≥ 2.0 mm or drop in systolic blood pressure ≥ 20 mmHg.

PeakVO_2_ was defined as the highest averaged VO_2_ during the last stage of exercise. Percentage values of predicted peakVO_2_ were calculated using the Wassermann formula,^18^ knowing the formula might underperform in HFpEF.^19^ VE/VCO_2_ slope was calculated to estimate the ventilator response to exercise. Values for peakVO_2_ < 14 mL/kg per minute and VE/VCO_2_ > 34 were considered to be abnormal based on the thresholds used for clinical risk prediction in patients with established HFrEF.^4^, ^20^, ^21^ The maximal expiratory exchange ratio (RER) was calculated as the VCO_2_/VO_2_ ratio during the last stage of exercise before recovery. A RER > 1.00 was considered index of maximal exercise.

#### Echocardiography

All study patients underwent comprehensive 2D echocardiography at rest using high‐end ultrasound systems (Philips EPIQ 7, Philips Medical Systems, Andover, MA). Echocardiography was performed in conditions of respiratory (<20 breaths/min), haemodynamic (systolic blood pressure 90–140 mmHg) and electrical (51–99 b.p.m.) stability. All sonographers were trained in accordance with a pre‐specified standard operation procedure. All 2D, Doppler and strain measurements were performed offline, at the echocardiographic core laboratory, using a customized software package (TomTec Image Arena, Unterschleissheim, Germany). All analyses were performed according to ASE/EACVI recommendations by a single investigator, with over‐reading by a second investigator. Both researchers were blinded to the clinical characteristics of the patients.

#### Blood samples

All blood samples were obtained between 09:00 am and 12:00 pm. All biomarkers were measured using established commercial assays.

### Endpoints and follow‐up

The total number of hospitalizations due to heart failure worsening or death was selected as the primary clinically relevant endpoint. Admissions due to heart failure worsening were considered as those occurring due to acute decompensated heart failure, acute pulmonary oedema, and cardiogenic shock, requiring intravenous diuretics and/or inotropic agents/vasopressors. Hospitalizations were identified from the clinical records of patients in the outpatient unit and hospital wards and from electronic medical records. Fatal events were identified from the clinical records of the outpatient unit, hospital wards, emergency room, and general practitioners and by contacting the patients' relatives. All patients included were followed up until December 2020.

### Statistical analysis

Continuous and categorical variables are presented as the mean ± standard deviation, median [interquartile range] or percentages, as appropriate. Depending on the distribution, Student's *t* test or Mann–Whitney *U* and chi‐squared test were used for analysis of continuous and categorical variables. The prognostic value of exercise parameters variables were determined using the Cox proportional‐hazards linear regression model. Univariate Cox regression analysis was used to determine the prognostic ability of peakVO_2_ and VE/VCO_2_ slope. Kaplan–Meier analyses with corresponding hazard ratio calculations were performed with the threshold values. The log‐rank test was used to compare of survival curves in the Kaplan–Meier analyses. Finally, for each potential combination of peakVO_2_ and VE/VCO_2_ slope, the mortality probability of the 99 patients whose results most closely resembled that combination was determined and plotted. All tests were two‐tailed. A *P* value < 0.05 was considered statistically significant. Analyses were performed using R version 4.2.3 (The R Foundation for Statistical Computing, 2023).

## Results

### Population characteristics

A total of 99 patients recruited in the German HFpEF Registry from August 2016 until May 2019 formed the study population. Mean age was 75.07 ± 7.31 years; 48 (49%) were female, 75 (75%) were in New York Heart Association functional class II, and the median [interquartile range] for N‐terminal pro‐B‐type natriuretic peptide (NTproBNP) was 511 pg/mL [287–906]. Comorbidities were common, especially arterial hypertension, dyslipidaemia, obesity and ischaemic heart disease. Nineteen patients (19%) were in atrial fibrillation. The remaining baseline characteristics of the cohort are summarized in *Table* *1*.

**Table 1:** Patient demographic and clinical characteristics according to CPET performance.

Variables	Included patients (*n* = 99)	PeakVO_2_ < 14 mL/min/kg (*n* = 39)	PeakVO_2_ ≥ 14 mL/min/kg (*n* = 60)	*P* value	VE/VCO_2_ slope > 34 (*n* = 40)	VE/VCO_2_ slope ≤ 34 (*n* = 59)	*P* value
Demographic							
Age, year	75.1 ± 7.3	76.8 ± 5.8	74 ± 8	0.058	76.4 ± 6.3	73.1 ± 8.3	0.026
Female	48 (49%)	26 (67%)	22 (37%)	0.004	29 (49%)	19 (48%)	>0.999
BMI, kg/m^2^	28.9 ± 5.2	31.6 ± 5.5	27.1 ± 4.1	<0.001	28.7 ± 4.9	29.1 ± 5.6	0.682
Medical history							
Hypertension	93 (94%)	38 (97%)	55 (92%)	0.398	56 (95%)	37 (93%)	0.683
Dyslipidaemia	69 (70%)	31 (79%)	38 (63%)	0.118	39 (66%)	30 (75%)	0.381
Diabetes mellitus	39 (39%)	21 (54%)	18 (30%)	0.022	24 (41%)	15 (38%)	0.835
Current smoker	12 (12%)	3 (8%)	9 (15%)	0.208	7 (12%)	5 (12%)	0.509
Previous smoker	42 (42%)	15 (38%)	27 (45%)	0.384	21 (36%)	21 (52%)	0.084
Ischaemic heart disease	55 (56%)	22 (56%)	33 (55%)	>0.999	33 (56%)	22 (55%)	>0.999
Atrial fibrillation	19 (19%)	6 (15%)	13 (22%)	0.603	9 (15%)	10 (25%)	0.299
Baseline NYHA class II	75 (75%)	23 (59%)	52 (87%)	0.001	42 (71%)	33 (83%)	0.621
Baseline NYHA class III	24 (24%)	16 (41%)	8 (13%)	0.003	17 (29%)	7 (18%)	0.238
CV admission within last year	56 (57%)	22 (56%)	34 (57%)	>0.999	35 (58%)	21 (52%)	0.540
HFpEF algorithms							
HFA‐PEFF score	5.5 ± 0.6	5.6 ± 0.6	5.5 ± 0.6	0.380	5.6 ± 0.5	5.4 ± 0.7	0.119
Vital signs							
Systolic blood pressure, mmHg	136.3 ± 20.4	136.3 ± 18.3	136.3 ± 21.9	0.997	135.4 ± 17.7	137.6 ± 24.1	0.144
Heart rate, b.p.m.	68.7 ± 13.1	69.8 ± 13.8	67.9 ± 12.7	0.480	69.5 ± 13.5	67.5 ± 12.4	0.460
*Laboratory*							
Haemoglobin. g/day	13.3 ± 1.3	13.1 ± 1.3	13.5 ± 1.4	0.207	13.2 ± 1.3	13.5 ± 1.4	0.351
eGFR, mL/min/m^2^	64.3 ± 18.8	56.1 ± 18.3	69.5 ± 17.4	<0.001	62.6 ± 19.8	66.7 ± 17.2	0.295
NTproBNP, pg/mL	511 [287–906]	612 [406.5–988]	437.5 [250–801]	0.037	619 [388.5–1022.5]	407 [249.3–645.5]	0.005
Treatment							
Beta‐blockers	83 (84%)	32 (82%)	51 (85%)	0.782	50 (85%)	33 (82%)	0.787
ACE inhibitors	38 (38%)	15 (38%)	23 (38%)	>0.999	26 (44%)	12 (30%)	0.207
ARB	48 (48%)	18 (46%)	30 (50%)	0.837	28 (47%)	20 (50%)	0.840
CCB	37 (37%)	18 (46%)	19 (32%)	0.202	22 (37%)	15 (38%)	>0.999
Aldosterone antagonists	2 (2%)	0 (0%)	2 (3.3%)	0.518	2 (3%)	0 (0%)	0.514
Loop diuretics	46 (46%)	25 (64%)	21 (35%)	0.007	26 (44%)	20 (50%)	0.682
Thiazides	24 (24%)	11 (28%)	13 (22%)	0.480	15 (25%)	9 (22%)	0.814
Statins	60 (61%)	24 (62%)	36 (60%)	>0.999	36 (61%)	24 (60%)	>0.999
Clinical follow‐up							
HF hospitalization	24 (24%)	11 (28%)	13 (33%)	0.480	19 (32%)	5 (13%)	0.032
Cardiovascular death	4 (4%)	2 (5%)	2 (3%)	0.412	4 (7%)	0 (0%)	0.069
Composite endpoint	24 (24%)	11 (28%)	13 (33%)	0.480	19 (32%)	5 (13%)	0.032

Abbreviations: ACE, angiotensin‐converting enzyme inhibitors; ARB, angiotensin receptor blockers; BMI, body mass index; CCB, calcium channel blockers; CV, cardiovascular; eGFR, estimated glomerular filtration rate; HF, heart failure; NTproBNP, N‐terminal pro‐B‐type natriuretic peptide; NYHA, New York Heart Association Classification.

When subjected to the recently developed HFA‐PEFF score to improve the diagnostic accuracy for HFpEF,^14^ our cohort confirmed to be well defined by obtaining a mean HFA‐PEFF score of 5.5 ± 0.6 points.

Mean ± standard deviation for peakVO_2_ and VE/VCO_2_ slope were 15.09 ± 4.75 mL/min/kg and 36.05 ± 6.6. Clinical characteristics of patients divided according to peakVO_2_ and VE/VCO_2_ slope are shown in *Table* *1*. Patients with peakVO_2_ < 14 mL/min/kg were more often female, had a greater BMI, had more diabetes, were more symptomatic (NYHA class III vs. II), had lower values of eGFR_MDRD_, had higher values of NTproBNP and were more often on loop diuretics. Patients with poorer CPET performance according to VE/VCO_2_ slope were older and presented higher NTproBNP values. The echocardiographic and CPET performance characteristics of the above‐mentioned subgroups of patients are recorded in *Table* *2*. Patients with peakVO_2_ < 14 mL/min/kg had a higher E/e′ ratio, had a lower heart rate and blood pressure at peak effort and achieved a lower maximum workload and RER. Patients with VE/VCO_2_ slope > 34 also achieved a lower maximum workload and had a greater V_D_/V_T_ at peak effort while showing no other significant differences. It should be noted that just 59 (60%) of our patients reached a RER > 1. When asked about the rate of perceived exertion, the subjects scored a mean of 15.8 ± 1.6 (hard until very hard) in the Borg scale. There were no serious adverse events during CPET.

**Table 2:** Patient echocardiographic and CPET characteristics according to CPET performance.

Variables	Included patients (*n* = 99)	PeakVO_2_ < 14 mL/min/kg (*n* = 39)	PeakVO_2_ ≥ 14 mL/min/kg (*n* = 60)	*P* value	VE/VCO_2_ slope > 34 (*n* = 40)	VE/VCO_2_ slope ≤ 34 (*n* = 59)	*P* value
Echocardiography							
LVEF, %	56.9 ± 5.2	56.9 ± 5.1	56.8 ± 5.3	0.925	56.7 ± 5.6	57.2 ± 4.7	0.654
LVMI, g/m^2^	108.7 ± 27.7	102.7 ± 27.2	112.7 ± 27.4	0.078	108.5 ± 26.5	109.1 ± 29.6	0.912
LAVI, mL/m^2^	44.3 ± 17.3	44 ± 19.3	44.6 ± 16.0	0.871	46.9 ± 19.2	40.5 ± 13.2	0.068
e′, cm/s	6.8 ± 1.6	6.9 ± 1.4	6.7 ± 1.7	0.687	6.9 ± 1.6	6.7 ± 1.6	0.523
E/e′ ratio	13.9 ± 5.2	15.8 ± 5.7	12.7 ± 4.5	0.004	14.2 ± 5.6	13.6 ± 4.6	0.578
CPET							
Atrial fibrillation	18 (18%)	6 (15%)	12 (20%)	0.606	10 (17%)	8 (20%)	0.792
Atrial paced rhythm	6 (6%)	3 (8%)	3 (5%)	0.678	5 (8%)	1 (3%)	0.397
Ventricular paced rhythm	8 (8%)	4 (10%)	4 (7%)	0.708	5 (8%)	3 (8%)	>0.999
Heart rate at rest, b.p.m.	70.5 ± 12.6	73.3 ± 15	68.7 ± 10.6	0.077	71.1 ± 13	69.5 ± 12.2	0.530
Heart rate at peak, b.p.m.	107.7 ± 24.7	95.4 ± 22.9	115.7 ± 22.7	<0.001	107.3 ± 21.3	108.3 ± 29.3	0.834
Systolic blood pressure at rest, mmHg	125.5 ± 19.1	127.3 ± 17.4	124.4 ± 20.1	0.481	124.8 ± 19	126.6 ± 19.4	0.648
Diastolic blood pressure at rest, mmHg	72.6 ± 11.3	71.9 ± 12.4	73 ± 10.6	0.664	71.8 ± 10.7	73.9 ± 12.2	0.380
Systolic blood pressure at peak, mmHg	167.4 ± 29.2	156.5 ± 26.9	174.8 ± 28.6	0.004	168.5 ± 28.3	165.6 ± 30.8	0.657
Diastolic blood pressure at peak, mmHg	75.9 ± 16.5	70.9 ± 14.5	79.3 ± 17.1	0.020	73.8 ± 15.4	79.1 ± 17.9	0.148
Workload, W	85.9 ± 33.2	62.6 ± 23.3	101.1 ± 29.7	<0.001	79.7 ± 31.1	95.1 ± 34.4	0.023
RER at peak	1 ± 0.09	0.94 ± 0.09	1.03 ± 0.07	<0.001	1 ± 0.08	0.99 ± 0.1	0.551
Borg RPE scale	15.8 ± 1.9	16.2 ± 2	15.6 ± 1.8	0.098	16.1 ± 1.8	15.5 ± 2	0.145
V_D_/V_T_ at peak	17 ± 3.4	17.2 ± 3.1	16.8 ± 3.7	0.602	18.2 ± 3.1	15.2 ± 3.1	<0.001

Abbreviations: CPET, cardiopulmonary exercise testing; LAVI, left atrial volume index; LVEF, left ventricular ejection fraction; LVMI, left ventricular mass index; RER, respiratory exchange ratio; RPE, rate of perceived exertion; V_D_/V_T_, dead space ventilation/tidal volume.

The patients were followed up for a median of 30 months [interquartile range, 24–38.5]. During follow‐up, 4 patients died, and 68 patients were hospitalized; thereof, 50 patients had cardiovascular hospitalizations. From these, 24 patients were hospitalized due to heart failure. In total, there were 207 all‐cause hospitalizations, 126 cardiovascular hospitalizations, 58 heart failure hospitalizations and 4 deaths.

The primary outcome event occurred in 11 of 39 patients (28.2%) with a peakVO_2_ < 14 mL/min/kg and in 13 of 60 patients (21.7%) with a peakVO_2_ ≥ 14 mL/min/kg [hazard ratio: 1.34; 95% confidence interval (CI): 0.60–2.99; *P* = 0.47]. During follow‐up, 5 of 40 patients (12.5%) with a VE/VCO_2_ slope ≤ 34 and 19 of 59 patients (32.2%) with a VE/VCO_2_ slope > 34 registered a primary outcome event [hazard ratio, 2.69; 95% confidence interval (CI): 1.00–7.21; *P* = 0.04]. This univariate Cox regression analysis showed that only VE/VCO_2_ slope was a significant predictor of both heart failure related hospitalization and mortality (see *Table* *3*).

**Table 3:** Univariate Cox regression analyses for peakVO_2_ (</≥14 mL/min/kg threshold) and VE/VCO_2_ slope (≤/>34 threshold).

	Events	HR (95% CI)	*P* value
PeakVO_2_ < 14 mL/min/kg	Mortality/HF hospitalization	1.34 (0.60–2.99)	0.47
VE/VCO_2_ slope > 34	Mortality/HF hospitalization	2.69 (1.00–7.21)	0.04

Abbreviations: CI, confidence interval; HF, heart failure; HR, hazard ratio.

In the multivariate analysis, after adjusting for gender, only VE/VCO_2_ slope was independently associated with the risk of admission due to heart failure worsening or death (*Tables* *4* and *5*).

**Table 4:** Multivariate Cox regression analyses for peakVO_2_ (</≥14 mL/min/kg threshold).

	Events	HR (95% CI)	*P* value
PeakVO_2_ < 14 mL/min/kg	Mortality/HF‐hospitalization	1.42 (0.62–3.27)	0.40
Female	Mortality/HF‐hospitalization	0.79 (0.34–1.81)	0.58

Abbreviations: CI, confidence interval; HF, heart failure; HR, hazard ratio.

**Table 5:** Multivariate Cox Regression Analyses for VE/VCO2 slope (≤/>34 threshold).

	Events	HR	*P* value
VE/VCO_2_ slope > 34	Mortality/HF hospitalization	2.73 (1.02–7.31)	0.04
Female	Mortality/HF hospitalization	0.82 (0.37–1.83)	0.62

**Figure 1:**
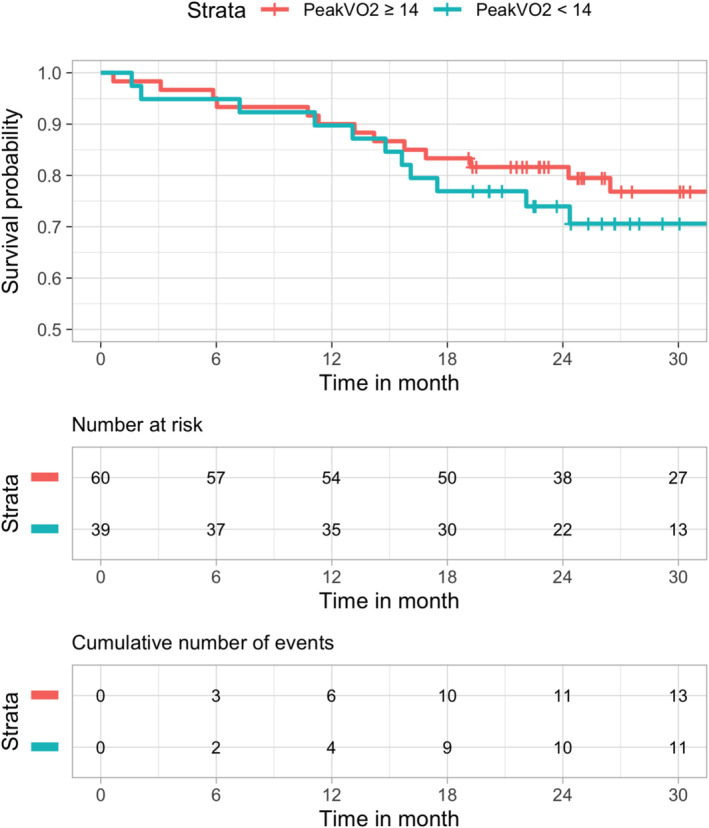
Kaplan–Meier survival curve according to peakVO_2_ (</≥14 mL/min/kg threshold).

**Figure 2:**
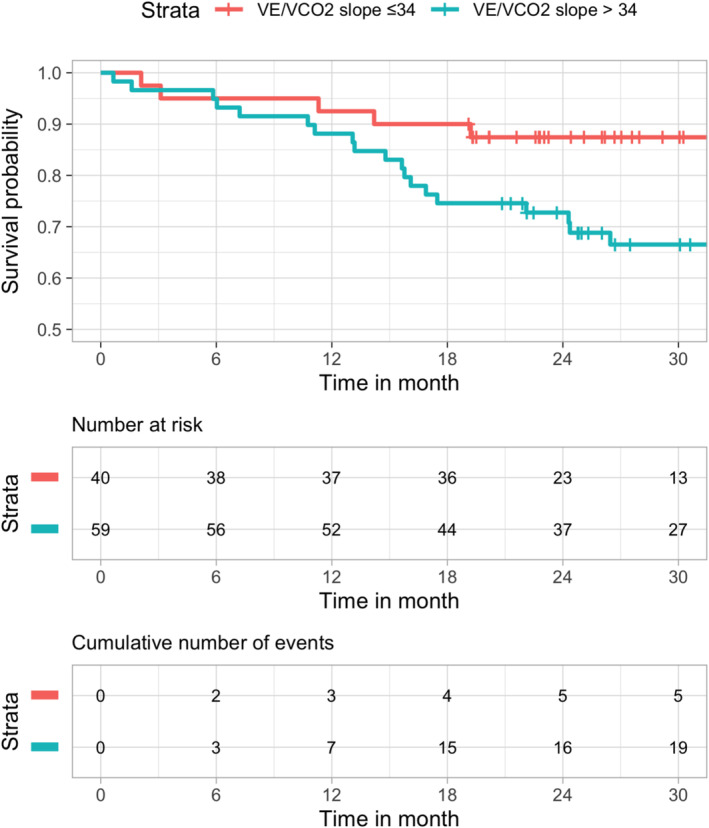
Kaplan–Meier survival curve according to VE/VCO_2_ slope (≤/>34 threshold).

Kaplan–Meier curves for the combined end point heart failure associated hospitalization and mortality using peak VO_2_≥/<14 mL/min/kg and VE/VCO_2_ slope>/≤34 as predictors are shown in *Figures* *1* and *2*.

## Discussion

The prognostic significance of the CPET parameters peakVO_2_ and VE/VCO_2_ slope in patients with HFrEF is widely accepted^4^, ^20^, ^21^, ^22^, ^23^, ^24^ with VE/VCO_2_ slope emerging as an often superior predictor of event‐free survival relative to peakVO_2_ in the recent years.^8^, ^21^, ^25^, ^26^, ^27^ The heterogeneity of the population with HFpEF, together with the novelty on its diagnosis, makes the evidence regarding the prognostic value of the CPET parameters in this cohort scarce. Previous reports delivered diverging conclusions regarding the prognostic value of peakVO_2_ and VE/VCO_2_ slope in the HFpEF population.^5^, ^23^ These reports have therefore proposed new CPET derived variables, such as percent predicted maximum oxygen uptake,^5^ exertional oscillatory ventilation^24^ or percentage of predicted peak VO_2_.^6^ Recent studies on cardiopulmonary exercise testing in combination with stress echocardiography (CPET‐ESE) have brought exercise testing back to the spotlight, presenting it as an important tool on the diagnosis and prognostic stratification across the HF spectrum.^10^, ^11^, ^27^, ^28^ Also, cardiopulmonary exercise testing has been used as an end point in recent HF clinical trials.^29^, ^30^

To our knowledge, this is the first prospective study of CPET data in HFpEF patients as per current guidelines definition (symptoms and signs of HF, LVEF ≥ 50% and objective evidence of cardiac structural and/or functional abnormalities consistent with the presence of LV diastolic dysfunction/raised LV filling pressures, including raised natriuretic peptides^12^). The good definition of our cohort was ratified when subjecting it to the recently developed HFA‐PEFF score,^14^ with patients scoring a mean HFA‐PEFF score of 5.5 ± 0.6 points.

Our findings are concordant with those of Guazzi et al.^23^, ^31^ and Yan et al.^32^ and serve, due to their prospective character, as the validation of the prognostic value of VE/VCO_2_ in HFpEF patients as per current guidelines definition. The lack of peakVO_2_'s prognostic value in our cohort, extends to HFpEF patients the point made by Ponikowski et al. in HFrEF patients^33^: ventilatory inefficiency during exercise predicts mortality in HF even in the presence of a normal peak VO_2_. As such, an impairment in ventilatory efficiency should be considered a hallmark for the estimation of risk among the spectrum of patients with HF,^34^ being these risk estimates equally powerful among patients with reduced and preserved systolic function. From a physiological perspective, VE/VCO_2_ has been reported to be related with right ventricle‐pulmonary vascular function and cardiac output in individuals with HF.^8^, ^13^, ^35^, ^36^, ^37^ Recent findings extrapolate this association to populations at risk for HF,^38^ making the noninvasively measured VE/VCO_2_ a relevant parameter in the early detection of HF.

Making up for the lack of multivariate analyses in previous studies, our findings still showed a strong association between VE/VCO_2_ slope and prognosis in a multivariate setting in this already well‐defined HFpEF population.

Lastly, most studies examining the prognostic value of CPET have either not included hospitalization as a clinically relevant endpoint or included all cause hospitalization. With acute heart failure as the leading cause for hospitalizations in subjects aged >65 years,^39^ we consider having used heart failure associated hospitalization as our primary endpoint a strength of our study.

Previous studies and ours highlight the importance of CPET for risk stratification in patients with HFpEF. Discrepancies in the association between CPET parameters and prognostically relevant events may be attributed to the small sample size of the studies and differences in the definition of HFpEF patients.

The present findings emphasize the prognostic value of VE/VCO_2_ slope and its importance as a reliable and accurate surrogate endpoint in the evaluation of new therapeutic strategies in HFpEF.

## Study limitations

The main limitation of our study lies in its relatively small number of patients. By choosing a combined end point consisting of heart failure associated hospitalizations and death, we found our sample size to be adequate for drawing preliminary conclusions. Also, our cohort adequately represented sex differences and the wide range of disease severity.

Our study did not address the pathophysiology behind our findings. Further studies are needed to understand whether ventilatory inefficiency in HFpEF patients is due to uneven perfusion/ventilation matching or muscle deconditioning with early metabolic acidosis. As our study was carried out before the implementation of SGLT2‐inhibitor therapy of HFpEF patients, it would be interesting to see if a SGLT2‐inhibitor therapy may improve VE/VCO_2_ slope in these patients.

We acknowledge the possible limitations for being a single centre observational study, which may account for potential confounders, thereby our findings should be cautiously extrapolated.

## Conclusions

In HFpEF patients as per current guidelines definition, a VE/VCO_2_ slope > 34 was associated with the risk of heart failure hospitalization and/or death. The present findings emphasize the prognostic value of VE/VCO_2_ slope and its importance as a reliable and accurate surrogate endpoint in the evaluation of new therapeutic strategies in HFpEF.

## Conflict of Interest

All authors have read and approved submission of the manuscript and have no conflict of interest to disclose.
